# Challenges and Opportunities for Overcoming Dog Use in Agrochemical Evaluation and Registration^*^

**DOI:** 10.14573/altex.2302151

**Published:** 2023-03-08

**Authors:** Patricia L. Bishop, Susy Brescia, Rachel Brunner, Warren Casey, Kathleen Conlee-Griffin, Richard A. Currie, Jeanne Domoradzki, Michelle Embry, Maria Ines Harris, Thomas Hartung, Gina M. Hilton, Barry Hooberman, Brandall Ingle, Kyung-Jin Jang, Lewis Kinter, Caroline Krall, Joseph Leedale, Anna Lowit, Jyotigna Mehta, Elizabeth Mendez, Bob Mingoia, Eliana Munarriz, Lynea Murphy, Angela Myer, Antoniana Ottoni, Martina Panzarea, Monique Perron, Juan Pina, Deborah Ramsingh, Fiona Sewell, Jennifer Swanson, Yu-Mei Tan, Andrea Terron, Maria A. Trainer, Marize Campos Valadares, Steven Webb, Elizabeth Webb, Catherine Willett, Douglas C. Wolf

**Affiliations:** 1 Humane Society of the United States, Washington, DC, USA; 2 Health and Safety Executive, Liverpool, UK; 3 United States Environmental Protection Agency (EPA), Washington, DC, USA; 3a formerly in United States Environmental Protection Agency, Office of Chemical Safety and Pollution Prevention, Office of Pesticide Programs, Washington, DC, USA and currently in United States Environmental Protection Agency, Office of Chemical Safety and Pollution Prevention, Office of Pollution Prevention and Toxics, Washington, DC, USA; 4 National Institute of Environmental Health Services (NIEHS), Research Triangle Park, NC, USA; 5 Syngenta, Berkshire, UK; 6 Corteva Agriscience, Indianapolis, IN, USA; 7 Health and Environmental Sciences Institute (HESI), Washington, DC, USA; 8 Instituto Harris, São Paulo, Brazil; 9 Johns Hopkins University, Bloomberg School of Public Health and Whiting School of Engineering, Center for Alternatives to Animal Testing (CAAT), Baltimore, MD, USA; 10 University of Konstanz, CAAT-Europe, Konstanz, Germany; 11 PETA Science Consortium International, Stuttgart, Germany; 12 US Department of Health and Human Services, Washington, DC, USA; 13 Outer Biosciences, Palo Alto, CA, USA; 14 Green Lawn Professional Scientific Consulting, DE, USA; 15 Johns Hopkins University School of Medicine, Baltimore, MD, USA; 16 ADAMA, Reading, UK; 17 Universidad de Buenos Aires, Department of Biological Chemistry, Ciudad Autónoma de Buenos Aires, Argentina; 18 European Food Safety Authority, Parma, Italy; 19 Atanor S.C.A., Munro, Argentina; 20 Health Canada, Ottawa, ON, Canada; 21 National Centre for the Replacement Refinement & Reduction of Animals in Research, London, United Kingdom; 22 independent writer, USA; 23 Australian Pesticides and Veterinary Medicines Authority, Armidale, NSW, Australia; 24 Laboratory of Education and Research in In Vitro Toxicology (Tox In), Faculty of Pharmacy, Universidade Federal de Goiás, Goiânia, GO, Brazil; 25 Bayer Crop Science, Chesterfield, MO, USA; 26 Humane Society International, Washington, DC, USA; 27 Syngenta Crop Protection, Greensboro, NC, USA

## Abstract

Progress in developing new tools, assays, and approaches to assess human hazard and health risk provides an opportunity to re-evaluate the necessity of dog studies for the safety evaluation of agrochemicals. A workshop was held where participants discussed the strengths and limitations of past use of dogs for pesticide evaluations and registrations. Opportunities were identified to support alternative approaches to answer human safety questions without performing the required 90-day dog study. Development of a decision tree for determining when the dog study might not be necessary to inform pesticide safety and risk assessment was proposed. Such a process will require global regulatory authority participation to lead to its acceptance. The identification of unique effects in dogs that are not identified in rodents will need further evaluation and determination of their relevance to humans. The establishment of *in vitro* and *in silico* approaches that can provide critical data on relative species sensitivity and human relevance will be an important tool to advance the decision process. Promising novel tools including *in vitro* comparative metabolism studies, *in silico* models, and high-throughput assays able to identify metabolites and mechanisms of action leading to development of adverse outcome pathways will need further development. To replace or eliminate the 90-day dog study, a collaborative, multidisciplinary, international effort that transcends organizations and regulatory agencies will be needed in order to develop guidance on when the study would not be necessary for human safety and risk assessment.

## 1 Introduction

In 2011, the Johns Hopkins Center for Alternatives to Animals Testing (CAAT) hosted two workshops on the critical evaluation of the use of dogs in biomedical research and testing (Zurlo et al., 2011; Hasiwa et al., 2011; Turner, 2011). At these workshops, the attendees reviewed how data from laboratory studies conducted with dogs are used to evaluate human safety and risk concerns across the entire spectrum of medical research, drug development, and chemical evaluation. The results of the workshop identified opportunities to improve the lives of dogs while in the laboratory setting as well as the development of new research tools that had the potential to eliminate the need for using dogs as a model species to address human safety and risk.

The present workshop report focused on the use of the 90-day dog study (OECD TG 409^1^; OPPTS 870.3150^2^) for evaluation and registration of pesticidal agrochemicals, also known as plant protection products. In the intervening decade since the 2011 workshop, the speed and magnitude of research in developing new tools, assays, and approaches to address human safety and risk questions have made it possible to re-evaluate the necessity of toxicity data generated using dogs as an animal model in agrochemical research and development. In addition to advances in the science, significant changes in regulatory policies and determinations made on the data needed to inform risk management decisions regarding the safe use of crop protection chemicals have provided the opportunity to address this issue specifically for the agrochemical sector.

When registering a new pesticide active ingredient, most countries require toxicological data from numerous animal studies. The US Environmental Protection Agency (USEPA) has had a leading role in creating a structure whereby the necessity of a particular test guideline study for making a health-protective human risk determination can be assessed when evaluating new agrochemicals. Guidance issued by the USEPA allows companies to request a test guideline study be waived if a weight-of-evidence evaluation of available data can show that the study would not benefit the evaluation (USEPA, 2013). This waiver program has been highly successful, saving the lives of over 200,000 animals including hundreds of dogs (Craig et al., 2019). Retrospective analyses of the use of particular required studies in past pesticide evaluations have also demonstrated these studies are rarely used for human and ecological safety and risk assessments, so the USEPA has issued guidance to waive these studies (USEPA, 2016, 2020a,b). Recently, the USEPA has been prioritizing efforts to reduce the use of vertebrate animals for chemical evaluation in research and testing and has produced a workplan for developing and incorporating new approach methodologies (NAMs) into hazard and/or risk assessments with the view that these methods may eventually be used in lieu of animal tests (USEPA, 2021). In parallel with the USEPA, the European Food Safety Authority (EFSA) has proposed a strategy for moving toward the application of NAMs for regulatory decision-making^3^.

These efforts are consistent with the global movement to establish NAMs for replacing the traditional whole animal testing. Several NAMs have been validated and applied across many chemical sectors, including agrochemical evaluation, and are now accepted OECD test guidelines^4^. In support of these dramatic movements away from the extensive use of animals in chemical testing, several internationally-recognized organizations including CAAT, are moving ahead in developing, evaluating, and qualifying alternative assays and models that provide data equivalent to or better than animal tests for informing human safety and risk^5^.

Historically, most pesticide regulatory authorities required both subchronic 90-day and chronic one-year oral studies in dogs for successful registration of a new active substance in crop protection products. The one-year dog study (OECD TG 452^6^) was removed from the list of required studies by all regulatory authorities by 2018 after multiple retrospective analyses comparing the results of the chronic and subchronic studies (e.g., Spielmann and Gerbracht, 2001; Dellarco et al., 2010; Kobel et al., 2010, 2014; Linke et al., 2017) found that additional exposure time did not change the risk-based conclusions determined with the 90-day study alone. Now the one-year dog study is listed as a conditional requirement and no longer routinely conducted as part of pesticide registration packages, saving the lives of hundreds of dogs annually.

The present workshop was convened to address the need for continuing to conduct the 90-day dog study for registration of a new active substance in a crop protection product. The workshop organizing committee established a problem formulation statement following the framework of Sauve-Ciencewicki et al. (2019). The agreed-upon problem statement was that *the 90-day dog study was being conducted for agrochemical authorization when it may not always be needed to adequately address hazard identification and human safety and risk.* A virtual workshop, *Challenges and Opportunities for Overcoming Dog Use in Agrochemical Evaluation and Registration* was held to discuss the role of the 90-day dog study in global regulatory decisions on agrochemicals and potential strategies for substantially reducing its use. The workshop was held as a two-part event, comprised of a series of presentations offered through a public information and question session on October 25, 2021, and a subsequent, invitation-only workshop on November 8–10, 2021.

## 2 Public workshop

The public workshop began with a welcome and introduction from **Anna Lowit, PhD,** USEPA, and **Andrea Terron, DVM**, EFSA. Background was provided on the removal of the requirement for the chronic (one-year) dog study through independent undertakings by several global researchers and regulatory agencies over the past 20 years and current efforts by the USEPA to reduce the use of laboratory animals, including dogs, for chemical safety testing. Remarks were also provided on the challenges and opportunities for reducing dog use in agrochemical evaluation and registration. Completely eliminating the use of dogs in pesticide testing is a major challenge. This workshop and any associated initiatives that arise from these meetings should be based on scientific reasoning, including experience from the last two decades, to guide future research and determine relevance and necessity of animal use. Regarding regulatory acceptance, key questions must be considered, including whether a second species is actually used to inform regulatory decision making, whether the experimental design of the study (four dogs/sex/dose group) sufficiently accounts for interspecies/intraspecies variability, and whether the dog has unique characteristics that can add value in chemical risk assessment, particularly for agrochemicals. To help answer these questions, retrospective analyses should be conducted to better understand the role of dog studies in agrochemical risk assessment. Efforts should focus on identifying critical research questions, evaluating the cost/benefit of animal use, shifting the paradigm from observation of apical endpoints to mechanistic understanding, using human-relevant systems to evaluate biological processes, and allocating more resources to identify the underlying mechanisms of toxicity, not just the toxicity endpoint itself.

The workshop progressed with several presentations, which can be found in full on the CAAT YouTube Channel^7^ and are briefly summarized below:

**Lewis Kinter**, **PhD,** GLP Scientific Consulting, presented the *Origins, History, Utility, and Future of Canine Bioassays in Pharmaceuticals R&D*, which provided an overview of the origins of dog use in pharmaceutical R&D, trends in dog use in the US and United Kingdom (UK), challenges and considerations related to bioassay-based animal research, regulatory guidelines on dog safety bioassays and use of data from these studies in decision-making, and overall contributions of dog safety bioassays. According to data from US Department of Agriculture (USDA), annual dog use has been declining from approximately 200,000 dogs in 1973 to approximately 60,000 in 2017 (NASEM, 2020). The primary driver for the decrease in dog use in the latter 20^th^ century was a paradigm shift from traditional animal bioassay/phenomenological endpoints to modern cellular/molecular biology and molecular endpoints. Currently, the use of dogs in biomedical research is predominantly for purposes of regulated product development and satisfaction of regulatory requirements for pharmaceutical, industrial, and agrochemicals. Most dogs were used by research organizations and private companies engaged in product development activities (NASEM, 2020). When animals are used in research, the difference between hypothesis- and bioassay-based animal research must be considered, recognizing the unique characteristics of animal bioassays, including factors that may influence outcomes of these studies (e.g., test article, research animal, research staff, and environment). The history of animal research regulation in the US was discussed, including how, due to differences between requirements of the US Food and Drug Administration (US FDA) and those of other global regulatory agencies, additional animals were often needed to meet the requirements of agencies worldwide. This stimulated the development of the International Conference on Harmonization of Technical Requirements for Registration of Pharmaceuticals for Human Use (ICH) in 1990, which outlines the requirements for bioassays used to identify safety concerns and appropriate dosing for clinical trials of pharmaceuticals. Use of rodent and non-rodent species (including dog) in preclinical testing is covered under the M3 guidance, which defines the goals of and justification for canine safety bioassays (US FDA, 2010). The canine bioassay has made contributions to science and human and veterinary medicine over time, and although not predictive of cross-species translation, it continues to contribute to decision-making in pharmaceutical R&D and human and veterinary medicine.

**Patricia L. Bishop**, **MS,** The Humane Society of the United States, presented *The Value of the 90-Day Dog Study for Pesticide Registration Toxicity Testing in the United States*. The talk provided an overview of testing requirements for registering a new pesticide active ingredient in the US, details of and rationale for performing the 90-day dog study, factors that may explain observed greater sensitivities in dogs compared with other species, and results of a retrospective analysis of 164 U.S. EPA pesticide human health risk assessments prepared in approximately the last 20 years to determine the value of the 90-day dog study in pesticide hazard identification and risk assessment.

Testing requirements for a new pesticide active ingredient follow the US Code of Federal Regulations Title 40, Part 158 (US Federal Register, 2022), and vary depending on pesticide use (food vs non-food), exposure route (oral, inhalation, and dermal), and duration (acute, subchronic, chronic). The subchronic 90-day non-rodent (usually dog) study is a standard test required for pesticide registration and is typically conducted in addition to the required subchronic 90-day study in rats. Testing in the dog is done ostensibly to reveal unique toxic effects that may not have been observed in the rodent or toxic effects at lower doses than observed in the rat indicating that the dog may be a more sensitive species and thus more appropriate for determining human safety and risk.

In an analysis of human health risk assessments prepared in approximately the last 20 years by the USEPA for new pesticide active ingredients submitted for registration, a number of findings support that the 90-day oral dog study was not a substantial contributor to human safety and risk determination for most pesticides, and thus had minimal value for a registration decision. In some cases, the apparent greater sensitivity in the dog, defined as having the lowest no-observed-adverse-effect-level (NOAEL) when compared to other animal studies of similar duration and route of exposure, could be explained by how doses were selected and spaced in the study. In other cases, when dog NOAELs were compared to other study NOAELs in rodents and rabbits using allometric scaling, a mathematical conversion that normalizes doses based on species differences in body mass and metabolism (Kleiber, 1932, 1947, 1961; Bokkers and Slob, 2007), the NOAELs from other studies were actually the same as or lower than those in the dog, indicating that in these cases the dog was not more sensitive to treatment effect. The analysis concluded that, for most of the pesticides for which the dog study was used in risk assessment, it was not necessary to support human safety and risk decisions and an equivalent, protective decision on safe use of the agrochemical could have been made had the dog study not been performed. Recommendations included conducting further analysis on the need for dog studies and development of criteria for issuing waivers.

**Martina Panzarea, MS**, EFSA, presented *Relevance of Dog Studies for the Derivation of Health-based Guidance Values for Plant Protection Products Approval — EFSA*. The presentation provided an overview of European Union (EU) requirements for regulatory submission of plant protection product active substances and data from a retrospective analysis intended to define the utility of dog studies to adequately assess safety of these active substances. Under the Commission Regulation (EU) 283/2013 (EC, 2013), these active substances must undergo oral toxicity testing in rodents (90-day rat study) and non-rodents (90-day dog study). Of note, although the one-year dog study is no longer a requirement for registration, this study remains available for a large proportion of substances under the authorization renewal process due to it being performed before most countries eliminated it as a required study. Eight animals (four females and four males) per dose group are required by OECD TG 409 (repeated dose 90-day oral toxicity study in non-rodents). Subchronic and chronic toxicity studies are used to derive dietary reference values. The NOAEL for the most sensitive human-relevant toxicologic parameter, normally in the most sensitive species of experimental animals, is used as the starting point to derive dietary acceptable daily intakes (ADIs) and set the basis for consumer risk assessment. A safety factor that takes into consideration the type of effect, the severity or reversibility of the effect, and inter- and intraspecies variability, is applied to the NOAEL to determine the ADI for humans. In the retrospective analysis, 432 EFSA conclusions and EU Commission Review Reports issued between 2001 and 2020 were screened for information on the most sensitive species selected to derive the ADI. For many of these substances, kinetic variability, allometric scaling, and dose spacing can explain differences in NOAELs observed between dogs and rodents. Although some hazards (cardiac, ocular, hematologic) would likely have been missed in the absence of dog studies, there were few cases identified in which this had an impact on the selection of the ADI. Comparing EFSA data with USEPA agrochemical data from the Toxicity Reference Database (ToxRefDB), version 2014 (USEPA, 2018), showed that EFSA and ToxRefDB data were similar in 60% of analyzed cases, which could help identify a harmonized approach for reducing/eliminating dog use in agrochemical evaluation and registration. Findings from this evaluation suggest that the dog is not always useful as part of the process of agrochemical risk assessment. A clear decision strategy developed for determining when the dog is necessary for the assessment based on critical physiological similarities to humans is warranted. Comparative *in vitro* metabolism studies now required as part of EU plant product evaluation are expected to add evidence regarding the relevance of using the dog as a necessary step in risk assessment.

**Lynea Murphy**, **PhD**, Corteva Agriscience, presented *Incorporating Toxicokinetic and Toxicity Data to Evaluate the Value Added from Using Dogs in Subchronic Toxicity Testing for Agrochemicals*. To determine species sensitivity using previously generated toxicokinetics (TK) and toxicity data, a retrospective analysis was conducted using an herbicide with the intent to provide some insight on how the available *in vivo* TK and systemic toxicity data would have informed selection of the appropriate species for human health risk assessment. TK evaluations can be integrated into short-term rodent and dog studies, enabling evaluation of dose proportionality, systemic exposure, and better characterization of the relationship between applied vs internal dose.

The *in vivo* rat and dog TK and toxicity data for the herbicide case example indicated that both species display sublinear kinetics along with no observed toxicity, up to the OECD defined limit dose of 1000 mg/kg bw/day. The study focused on the use and application of TK data for dose setting and interpretation because there was no toxicity. The parent compound of this herbicide is rapidly metabolized to a primary metabolite and the analysis was conducted on the metabolite. Thus, the acid metabolite was used as a dose metric. AUC_24hr_ data supported that overall systemic exposure to the primary metabolite (a hydrolysis product) is lower in the dog compared to the rat at similar exposure levels with no observed toxicity, thereby indicating that the rat could potentially be considered the more sensitive species for risk assessment.

The available data used in this retrospective analysis included single dose metabolism probe study data, along with repeat-dose TK and toxicity data, in addition to *in vitro* data evaluating unique species metabolism, microsomal clearance, and plasma protein binding. Part of this case example included the evaluation of using only *in vitro* and physical chemistry data to simulate systemic exposure data in the rat and dog. In this scenario, aqueous solubility and logP data, along with *in vitro* microsomal clearance and plasma protein binding data were used as data inputs into GastroPlus Software^8^ to determine if *in silico* and *in vitro* data alone could provide similar trends for systemic exposure compared to single dose *in vivo* TK data. The model predicted a higher systemic exposure to the primary metabolite in the rat, which was consistent with the available *in vivo* TK data and provided support that *in silico* and *in vitro* data can be used as an initial assessment of species TK differences.

This example is an important first step to better characterize the utility of multiple data streams in a hypothetical decision tree and to consider how a tiered approach using *in silico, in vitro,* along with *in vivo* data from short-term rodent and dog studies can provide a preliminary determination of the most sensitive, and therefore, most relevant species for human health risk assessment. The decision tree approach, which evaluates and integrates TK and toxicity data, can be used to refine testing strategies to include only the sensitive and relevant species for human health risk assessment, and potentially reduce unnecessary use of dogs for agrochemical toxicity testing.

**Kyung-Jin Jang**, **Ph.D.**, Emulate, presented *Species Liver-Chip to Assess Cross-Species Drug Toxicity and Human Relevance*. Approximately 90% of clinical trials fail to produce an approved therapy for human use (Zurdo, 2013; Harrer et al., 2019). Drug-induced liver injury (DILI) is the most frequent reason for withdrawal of drugs from the market. Poor prediction of DILI in humans is driven by poor preclinical-to-clinical translation, emphasizing the need for improved, physiologically relevant models to better predict human sensitivity to hepatotoxic drugs (Baudy et al., 2020; Monticello et al., 2017). Liver-Chip technology is a next-generation platform providing insight into human biology with which to study human physiology and diseases. The Liver-Chip is a dual-layer chip consisting of an upper and lower channel, separated by a porous membrane with the upper channel containing approximately 50,000 primary hepatocytes cultured between extracellular matrix layers. Nonparenchymal cells (liver sinusoidal endothelial, Kupffer, and stellate) are cultured in the lower channel. Cells in each channel can interact across the porous membrane. Unidirectional flow can be applied to the channels, each with a different medium, to provide a more physiologically dynamic environment within the chip. For drug testing and species comparison, compounds can be added to both channels or to either top or bottom channels to study drug transport, metabolism, and various reactions with cells within the chip. Examples of DILI response for three drugs were provided which illustrated species differences in hepatotoxicity and description of mechanisms of action to functional outcome. These findings demonstrate that data from species-based Liver-Chip studies closely emulate data from *in vivo* studies in the same species (Jang et al., 2019). In clinical development, Liver-Chip technology can be used for hepatotoxicity prediction, human risk assessment, mechanistic investigation, and biomarker discovery and provides insights into more complex biological questions. This technology has the potential to contribute to the reduction and eventual replacement of animal testing, including the use of dogs in agrochemical safety testing.

**Douglas C. Wolf, DVM, PhD.**, Syngenta Crop Protection, provided closing remarks for the public information session, summarizing the workshop goals and emphasizing both the importance of pesticides in protecting plant health for food, fuel, feed, fiber, and botanical pharmaceutical production and testing of these chemicals to assess impacts on human, animal, and environmental safety, being mindful of the need to reduce animal use in this testing and consider scientific validity when determining the most sensitive species for each test. The progress made to date in eliminating the one-year dog study was reiterated. The problem statement that *the 90-day dog study was being conducted for agrochemical authorization when it may not always be needed to adequately address hazard identification and human safety and risk* was reintroduced and, based on the presentations from the several speakers, one could come to the conclusion that there were numerous cases when the 90-day dog study could have been avoided while still reaching health protective decisions regarding safe use of a new crop protection product active ingredient. The challenge for the future is to come up with a framework or process to prospectively determine when the 90-day dog study can be avoided, resulting in saving the lives of many dogs without diminishing the protection of public health.

## 3 Invitation-only workshop

The invitation-only session of the workshop provided an opportunity to discuss next steps in assessing the value of the 90-day dog study for pesticide testing and human risk assessment with the eventual goal of establishing a decision framework for enabling the waiving of the 90-day dog study for agrochemical regulatory submission. Previous workshops held over a decade ago recommended the removal of the requirement of the one-year dog study in pesticide testing by all regulatory agencies (Hasiwa et al., 2011), an action that has since been adopted globally. As a lesson learned from this effort, it is important to engage all involved countries and regions, not just the US and EU, to achieve the eventual removal of the requirement for the 90-day dog study. The goal of this workshop was to involve attendees in discussions related to the 90-day dog study and develop action items to help facilitate progress in reducing or eliminating this study for pesticide testing. There were two breakout groups that focused on different, but related, topics: 1) strategies for eventually waiving the 90-day dog study, and 2) scientific technologies and approaches to inform species selection for toxicity testing.

### 3.1 Strategies for eventually waiving the 90-day dog study

This breakout session focused on strategies that could lead to waiving of the 90-day dog study. Topics of discussion included strengths and limitations of the retrospective analyes performed using data from USEPA and EFSA and opportunities to build on their conclusions and design future case studies to evaluate the value added from the dog study, elements that should be considered in an integrated decision tree for waiving the dog study and the need for global participation in further decision tree development, and lessons learned from the recent efforts to develop a framework for waiving the rodent bioassay requirement for carcinogenicity testing (Hilton et al. 2022). The discussion focused on using scientific approaches to determine if multiple species are needed in agrochemical risk assessment and if human health would be at risk without data from the 90-day dog study. Several questions were addressed including: Why are two species needed in subchronic studies and why was the dog chosen as the second species?

It was agreed that complete elimination of the 90-day dog study would be difficult at the present time and that the focus should be on establishing criteria for a waiver approach, which will require global agreement. As there were a few cases noted in the retrospective analyses where a dog study identified a potential health risk for humans missed by other studies, alternative approaches may be needed to confidently identify hazard and assess risk if the dog study were routinely waived. It is important to recognize that requirements for waiving or not performing a guideline study may be different according to the laws and regulations under which different regulatory bodies function. Efforts should be made early to harmonize requirements and regulations.

Although similar conclusions were reached from retrospective analyses, the methods used to reach the conclusions were different. Additional evaluation of the data using common approaches may be useful to further support the same conclusions.

Several ideas were considered worth pursuing, such as identifying the most sensitive species using human equivalent doses through the application of allometic scaling or benchmark dose (BMD) modeling, rather than basing on the lowest NOAEL. Also, the EFSA analysis could be expanded to evaluate the risks associated with occupational and residential exposure without the dog study, in addition to impacts already determined on the acceptable daily dietary intake. It will be important to identify any unique effects in dogs that are not seen in rodents, and when identified, determine if they are relevant for humans. There may be value in examining the extensive database from pharmaceutical studies for insights into human relevance (e.g., some pharmaceuticals appear to show cardiac effects relevant to humans in dogs). Patterns identified from previous studies comparing dog and human data can be useful to derive hypotheses for assessing for novel compounds through methods such as read-across. Examples of potential patterns are routes of administration, target organ effects, and chemical class or mode of action. Although many lessons can be learned from retrospective data analysis, it will likely not be sufficient to ultimately support the development of a framework to waive the dog study. Comprehensive prospective case studies will also be needed to identify and evaluate strategies for replacing the dog study.

The creation of a decision tree was seen as a useful first step leading to determining if the 90-day dog study can be waived. Application of the decision tree in case studies will provide information necessary to support a draft waiver guidance document. Such a document would need to be further evaluated across a broad audience of relevant experts and stakeholders for feedback. This kind of iterative effort would build evidence that could lead to decreasing the need for and potentially eliminating the 90-day dog study without or before changing legal requirements.

The original motivation of testing with both a rodent and a non-rodent species is to account for the possibility that the rodent species may not be sensitive to an effect to which humans are sensitive (Box and Spielmann, 2005). As more scientifically credible approaches are established, more mechanistic data are available to inform species sensitivity, allowing one to better answer whether the dog is or is not an appropriate species for human risk assessment. For example, *in vitro* metabolism data, *in vivo* TK and ADME studies, or potentially a short-term, non-lethal dog study, which may be sufficient or, alternatively, suggest that the 90-day dog study would be necessary. Thus, a stepwise approach should be taken to generate toxicological data and consult with regulatory agencies, rather than conducting all the required studies prior to submitting a registration data package and application.

The participants discussed the opportunity to adapt previously published frameworks and strategies as starting points for developing waiver criteria for the dog, focusing on human relevance, not specifically the dog (e.g., Hilton et al. 2022; Luijten et al. 2020). The evaluation and analysis of available legacy data and development of case studies will help to determine when the dog study was useful and when it did not contribute to safety or risk decisions. Such an effort could be advanced by setting up a workgroup to perform case studies, establish a decision tree for evaluating the needs for a subchronic dog study, and create a template for dog study waiver. For this effort to be successful in facilitating the regulatory agencies to re-evaluate the necessity of dog studies, regulatory authorities from Asia and South America should be included in this effort, in addition to the typical North American and European participants.

Conclusions from this breakout group included that publishing the retrospective analyses using data from USEPA and EFSA would make a significant contribution to ongoing efforts and provide a foundation for further investigation. Organization of a formal workgroup focused on a tiered decision tree for determining whether a dog study would be necessary, was recommended. This decision tree could then be shared with the international community for comment, refinement, and hopefully, acceptance.

### 3.2 Scientific technologies and approaches to inform species selection for toxicity testing

There is growing desire to minimize animal use in agrochemical toxicity testing and implement alternative methods and targeted shorter-term *in vivo* studies to optimize the design of longer-term animal studies, including appropriate species selection. *In vitro* approaches that can be used to provide critical data on relative species sensitivity, as well as human relevancy were discussed. The breakout group discussed what data from required studies or additional targeted studies could be used to determine whether a 90-day dog study would be useful in predicting human health risks for a particular chemical. An example of a scientific question to address is if the dog exhibits greater sensitivity for specific chemistry classes and whether the specific toxicology effect is relevant to humans. Further discussion addressed the specifics of information needed prior to considering a waiver for a subchronic dog study, and what tools are available or need to be developed to facilitate read-across evaluations. It is also important to develop strategies for reviewing and interpreting relevant information and identify alternative approaches that can provide equivalent or better toxicological information as the traditional dog guideline study. The mining of available information and development of a read-across database would be beneficial for those specific classes of chemistry where it has been shown that the 90-day dog study has not driven decisions regarding safety and risk assessment. Additional data reviews can identify under what circumstances the safety assessment are driven by NOAELs from the dog study. Case studies can then be designed to explore whether the observed toxicity endpoint is driven by a mechanism that is relevant to humans.

The participants discussed the types of studies and data that could be useful for determining if the 90-day dog study would add value to the safety evaluation of a chemical, such as *in silico* approaches for predicting toxicophores (parent molecule vs metabolite) and biologically-relevant dosimetry across species, biomarkers that are associated with early key events in a toxicity pathway, and transcriptomic analysis that can be used to identify modes of action and characterize dose-response.

Progress could be made more quickly as high-throughput assays are validated and to identify metabolites and key events in their agrochemical-induced mechanisms of action within biological systems leading to an adverse outcome pathway. The establishment of *in vitro* comparative metabolism models that would be able to determine the rate of metabolism, relevant enzymes, and species differences, was identified as a critical need. While the science progresses toward *in vitro* techniques, the use of short-term *in vivo* studies in rats, mice, and even dogs would be useful to better understand relevant biology that, when perturbed, could lead to a human-relevant adverse effect. Many of these quantitative approaches could be evaluated using BMD modeling to improve our assessment of dose-response, incorporating allometric scaling for improved dose comparison and interpretation of systemic exposure, and examining the role of dose spacing, to better clarify when the dog is the most sensitive or appropriate model.

There are some methods already available and fit-for-purpose that can be used to further evaluate the need for dog studies, such as physiologically based toxicokinetic modeling (PBTK), which, while a valuable tool, requires *in vivo* data to evaluate its predictive ability. A small set of models is ideal for use within a regulatory framework and selected models should be fully evaluated with respect to their capability.

## 4 Workshop conclusions

In order to achieve progress toward replacing or eliminating the 90-day dog study, there will need to be a collaborative, multidisciplinary, international effort that transcends organizations and regulatory agencies to develop guidance on when the study would not be used to inform human safety and risk assessment. Attendees acknowledged the extraordinary effort required to accomplish this goal and the workshop concluded with emphasis on the need to assemble leadership for this initiative with a request that action be taken to facilitate this work.

## Footnotes

1 https://www.oecd-ilibrary.org/environment/test-no-409-repeated-dose-90-day-oral-toxicity-study-in-non-rodents_9789264070721-en accessed February 23, 2022

2 https://www.regulations.gov/document/EPA-HQ-OPPT-2009-0156-0011 accessed February 23, 2022

3 www.efsa.europa.eu/sites/default/files/event/2020/Presentation-5.2.pdf accessed February 23, 2022

4 https://www.oecd.org/chemicalsafety/testing/oecdguidelinesforthetestingofchemicals.htm accessed February 23, 2022

5 https://ntp.niehs.nih.gov/iccvam/docs/roadmap/iccvam_strategicroadmap_january2018_document_508.pdf; https://www.uwindsor.ca/ccaam/; https://www.nc3rs.org.uk/ accessed February 23, 2022

6 https://www.oecd-ilibrary.org/environment/test-no-452-chronic-toxicity-s accessed February 23, 2022

7 https://www.youtube.com/playlist?list=PLq3BABaYKtzndvLCmE2NT5DkB_sfCNIUs accessed February 23, 2022

8 https://www.simulations-plus.com/software/gastroplus/pbpk-software/ accessed February 23, 2022
